# Training-Based Interventions in Motor Rehabilitation after Stroke: Theoretical and Clinical Considerations

**DOI:** 10.1155/2004/703746

**Published:** 2005-01-27

**Authors:** Annette Sterr

**Affiliations:** Department of Cognitive Neuroscience and NeuropsychologySchool of Human SciencesUniversity of SurreyGuildfordGU2 7XHUK

## Abstract

Basic neuroscience research on brain plasticity, motor learning and recovery has stimulated new concepts in neurological rehabilitation. Combined with the development of set methodological standards in clinical outcome research, these findings have led to a double-paradigm shift in motor rehabilitation: (a) the move towards evidence-based procedures for the assessment of clinical outcome & the employment of disablement models to anchor outcome parameters, and (b) the introduction of practice-based concepts that are derived from testable models that specify treatment mechanisms. In this context, constraint-induced movement therapy (CIT) has played a catalytic role in taking motor rehabilitation forward into the scientific arena. As a theoretically founded and hypothesis-driven intervention, CIT research focuses on two main issues. The first issue is the assessment of long-term clinical benefits in an increasing range of patient groups, and the second issue is the investigation of neuronal and behavioural treatment mechanisms and their interactive contribution to treatment success. These studies are mainly conducted in the research environment and will eventually lead to increased treatment benefits for patients in standard health care. However, gradual but presumably more immediate benefits for patients may be achieved by introducing and testing derivates of the CIT concept that are more compatible with current clinical practice. Here, we summarize the theoretical and empirical issues related to the translation of research-based CIT work into the clinical context of standard health care.

## 1. Introduction

Chronic motor deficits represent a common problem after brain damage such as stroke or traumatic brain injury. If the upper-limb is affected by the lesion (i.e. upper-limb hemiparesis) the deficits are especially obtrusive, and the condition often leads to disability and permanent dependency on community care. The effective treatment of chronic hemiplegia therefore represents a key aim in public health and neurorehabilitation research. Current treatment approaches are significantly influenced by the long-standing assumption that neural tissue in the central nervous system cannot regenerate and that the capacity for brain plasticity in the mature brain is very limited. As a result, treatments often aim to maximize the use of intact functions since the ultimate cause of the functional impairment cannot be cured [2,55]. In last two decades, however, basic neuroscience research in animals and humans has shed more light on the processes that enable the central nervous system to respond in an adaptive manner to injury, as well as the mechanisms involved in the re-acquisition of apparently lost behaviours [41]. This research has sparked the development of new, theoretically driven approaches in the treatment of motor deficits following acquired brain damage, which appreciate both the role of brain plasticity and the importance of practise in treatment-driven recovery of motor function ([6,14] for review [23,43,46,54,66–68,83,94,95]). The introduction of disablement models has further provided theoretical anchorage for the establishment of clinical outcome parameters and the evidence-based evaluation of treatment efficacy.

## 2. Neural correlates of motor recovery

Survivors of brain injury and stroke invariably show some degree of functional improvement over time.

ISSN 0953-4180/04/$17.00 © 2004 – IOS Press and the authors. All rights reserved

Such spontaneous recovery ranges from minimal to complete restoration [86]. In the 1960s, the Russian Neuropsychologist, Alexander Luria, proposed the idea that this recovery is facilitated by a reorganization of function in intact brain regions [49–51]. While Luria based his theory purely on clinical observations, more recent animal experiments confirm the initial hypothesis of reorganization after CNS lesions (e.g. [26,27, 34–36,56–58,61,69,97]). Human data obtained with non-invasive imaging techniques further suggests that the neural networks underlying primary representations undergo massive changes as a consequence of brain damage [15,22,98], and that these changes may be linked to perception and functional outcome [3,4,13, 18,19,64].

Neuroimaging experiments in chronic, well-recovered hemiplegic patients show movement-related activations in contralesional sensorimotor and premotor areas, the ipsilesional cerebellum, bilateral supplementary motor areas and the parietal cortex [10,12,70,92]. Most recently, Ward et al. [89,90] further demonstrated that affected arm movements induce distinguishable activation patterns for different recovery levels. Thus, chronic stroke patients with incomplete recovery activate a number of primary and non-primary motor regions over and above the activations seen in healthy controls. In patients with complete recovery, however, activations resemble those in healthy control participants. This suggests that the level of recovery is reflected in a functional reorganization of the neural circuits involved in controlling affected arm movements.

While these findings provide an important first step towards the understanding of spontaneous recovery, they do not answer the crucial question, i.e. what characterises the dynamics of changing brain activation patterns over time and what is their relationship with functional recovery. Regarding this question, Marshall et al. [53] and Calautti et al. [7] scanned stroke patients at one early and one late time point several months after the incident. In both studies, the initial activation in motor areas induced by affected arm movements was substantially stronger and more widespread as compared to the pattern found in the post-acute stage. This decrease in movement-related activation, however, was not correlated with the functional improvements made by patients. Thus, the reduction in task-related activation appeared to be a function of time rather than a function of recovery. Ward et al. [90] subsequently suggested that the reported lack of correlation between changed brain activation and functional outcome may not reflect a true absence of the expected link, but could be explained by the sparse capture of the recovery process and the relatively coarse outcome measures used to assess functional improvement. Henceforth, the authors conducted a longitudinal repeated-measures experiment in which patients were scanned more frequently and motor improvement was assessed with a comprehensive motor test battery. This experiment confirmed the previously reported decrease of activation magnitude across the various test sessions. Early after stroke, the brain activations were greater and more widespread, while at later stages, task-related activation in motor regions gradually decreased. Regarding *functional* recovery, the study further revealed a clear relationship between the reduction (or normalization) of motor-related brain activation and improved performance scores in individual patients.

The evolution of brain activation in recovering stroke patients, i.e. initial over-activation and subsequent down-regulation, is in line with results from animal studies [32,33,77]. These experiments have shown that dendritic branching and synapse numbers increase in the weeks following the lesion, while subsequently, these new connections are pruned back in an activity-dependent fashion. Furthermore, the activity-dependent fine-tuning of abundant synapses and dendritic connections is also observed when neural networks are established in the developing brain [40]. It appears therefore plausible to hypothesize that the observed activation pattern in recovering stroke patients reflects similar mechanisms. Alternatively, one could argue that higher and more widespread activations observed early in recovery are due to greater effort necessary for effecting the respective task. However since task difficulty was kept constant across recording sessions, this explanation appears less likely. We therefore conclude that the longitudinal study by Ward et al. (2003) suggests that performing a simple motor task initially draws on a widespread network of neurons that are activated in an unspecific manner. With increased functional recovery, motor control becomes more specific and thus the task-related activation abates. This relationship is correlational and does not allow for causal conclusions. However, we hypothesize that the abated activation reflects functional reorganization processes, which in turn result in better task performance. This might entail two intertwined processes, the activity-dependent pruning of surplus dendrites and synapses, and the stimulation-dependent optimisation of the neural networks involved in motor control. In other words, we presume that the brain ‘learns’ to control and execute the task in a more effective way by normalizing its activity levels and by optimising its neural circuitry. We further postulate that interventions, which involved affected arm training, shape the activity-dependent adaptation of the relevant neural networks in a functionally relevant fashion and that treatment-specific enhancements of recovery are effected via this mechanism. If this assumption is correct, the activity-dependent fine-tuning would appear to be crucial for functional outcome, and it is probably during this phase that the patient's behaviour/interventions have a particularly high impact.

## 3. The role of evidence-based approaches for the investigation of long-term motor recovery

In an ideal world, long-term recovery represents the re-gain/improvement of function that has been brought about by and is specific to a particular intervention. But current clinical practice in motor rehabilitation is often not evidence-based, and the majority of procedures/approaches presently employed reflect accepted practices and customs, the clinical efficacy of which remains an unproved assumption [88,91]. Furthermore, comparative studies on the efficacy of the various 'schools of intervention' (also termed 'approaches' [25]) have shown that none of those treatment methods is superior to the others with regard to the improvement of functional outcome, despite fundamentally different assumptions on treatment mechanisms [14,94].

One of the central developments in the conceptualisation of physical therapy and other rehabilitation professions lies in the introduction of disability models (e.g. ICD1-10 (WHO), 'Top-Down-Model of Rehabilitation' [20]) that provide a theoretical framework for clinical outcome research. In recent years, these models have supported a paradigm shift from descriptive efficacy studies at different model levels to a coherent research approach that provides *'direct evidence of the degree to which physical therapy that affects an impairment* (*e.g. muscle force*) *will also reduce disability and improve the functional outcome of the patient* (*e.g. in activities such as transfers, walking ability, and improved quality of life*)*'* [28] page 968). Combined with the development of set methodological standards in clinical outcome research (e.g. Consort Statement [96], the disability models and their testing provides the basis for evidence-based approaches in modern health care.

In line with these ideas, researchers have started to conduct small-scale randomized trials that are designed to test theoretically derived therapy components, as for example repetitive training of isolated movements [6] or EMG-induced muscle stimulation [9], in a systematic fashion. The results of these studies are generally encouraging and suggest that patients' performance improves following increased activation/stimulation interventions.

## 4. New treatment approaches stimulated by basic research: The example of constraint-induced (CI) therapy

CI therapy represents a treatment for chronic upper-limb hemiparesis that is rooted in basic brain research. It takes evidence from a wide variety of research fields into account and encompasses knowledge on the behavioural and the neuronal processes during spontaneous recovery, neuroplasticity and motor learning mechanisms, and the principles of behavioural therapy.

Designed to enforce the use of the affected arm, the intervention combines an unaffected arm constraint with affected arm training under massed practice conditions. These key principles are derived from deafferentation experiments in monkeys that have led to the discovery of the learned-non-use phenomenon (described in detail below [38,39,78–80]). The signature intervention is run over 12 consecutive days. Throughout this time period, the unaffected arm is constrained by a splint/sling device for 90% of waking hours, and movements with the affected arm are trained on the basis of shaping procedures for six hours each day [81]. *Shaping* refers to an operant conditioning method that is commonly used in animal learning experiments and behavioural therapy. The basic principle is to approach a behavioural objective (a specific movement with the affected hand in this case) in small steps of progressively increasing difficulty. Verbal feedback on task performance is given continuously and the slightest improvement is positively reinforced. By this means, the extended behavioural capacity is kept just beyond the performance already achieved. Controlled experiments and randomised clinical trials in chronic upper limb hemiparesis (chronicity $> 12$ months) have shown that CI therapy results in increased motor capabilities and an increased use of the affected hand in the real world environment [44,59,71,81,82,84].

## 5. Mechanisms that make CI therapy effective: Learned non-use & therapy-induced brain plasticity

Theoretically, CI therapy is centred around the learned-non-use hypothesis, which claims masked recovery of affected limb use. The initial phenomenon was discovered when researchers observed that monkeys, who had undergone upper-limb deafferentation, did not use their affected limb in the cage environment even though their motor abilities were nearly normal [38,39,45,87]. Subsequent experiments revealed that this 'non-use' represented an acquired behaviour that was due to instrumental learning during the spinal shock period, and, as a consequence of its learned origin, could be reversed by behavioural measures [78–80]. Making the additional assumption that a behavioural constellation similar to the spinal shock might be relevant during diaschisis, the treatment principles derived from the learned-use theory formed the basis of today's CI-therapy intervention. Initial evidence supports the idea of masked recovery in hemiplegic patients, i.e. that residual movement capabilities are not employed to fullest extend [1,74].

In addition to the learned non-use element, the efficacy of CI therapy is further promoted by neuroplasticity processes. Experimental research in animals and humans revealed that massed practice and positive reinforcement are necessary conditions to promote use-related adaptations of brain function [72 for review]. Recent studies on use-related plasticity and motor learning further suggest that enforced affected arm use supports the reconfiguration of neural representations, and thus enhances long-term recovery [29, 30,42,47,48]. Recovery reflects at least in part a regain/improvement of function, that is based on learning mechanisms. It follows from this argument that efficient motor rehabilitation interventions need to acknowledge the behavioural and neurobiological mechanisms of human learning and brain plasticity. Therapeutic interventions which enhance these processes may be therefore be more efficient [52,60,68]. The initial evidence on CI therapy and other training-based treatment concepts supports this idea.

## 6. Implementing training concepts in the clinical environment

Even though CI therapy has been shown to be effective, it is not fully accepted by clinicians and patients. Thus, a recent survey [62] of 280 stroke patients and 80 physical therapists revealed that 68% of patients were not interested in this treatment because of concerns about the practice schedule and the restrictive device. Clinicians further cited concerns about patient compliance and safety as well as the lack of resources to administer the intensive treatment. In addition, our own research activities leave no room to doubt that there are institutional reservations, mainly because the six-hour protocol conflicts with the modus operandi of the majority of rehabilitation providers. Thus, it is perceived that major organizational adjustments would be necessary to accommodate the application of the signature CI therapy intervention. Combined with the clinical concerns, this 'impracticality' represents a serious obstacle in bringing this undoubtedly effective intervention into standard care.

One way to address the issue of clinical transfer is to clarify the contribution of treatment intensity/massed practice as well as the unaffected arm constraint for treatment success. In a first step we therefore compared the clinical benefits obtained with a CI therapy protocol that employed reduced daily training intervals with the signature CI therapy intervention [71]. In this experiment all treatment factors, i.e. constraint, overall time of intervention, residual movement abilities at intake, chronicity etc, were kept constant except for the amount of daily shaping training, which was reduced by 50% to 3 hours a day in the amended protocol treatment group. The rationale for testing less daily training was two-fold. First, patients with poorer physical condition have often less capacity for demanding activities, and 6 hours of daily training may be too strenuous for them. In addition, it may also act against the therapy's effectiveness, when a patient is pushed beyond his/her endurance limits and becomes fatigued. Secondly, studying the effects of enrichment on the recovery from brain lesions in animals, Will et al. [93] found that enrichment of two hours a day was as beneficial as 24 hours a day, which raises the question about the optimal amount of training. This issue is of course most relevant for the 'practicality' and resources concerns mentioned above. The study employed a mixed design which comprised a group factor and the repeated measure of outcome parameters at baseline, pre- and post-treatment and weekly follow-up assessments for one month. The results revealed a pronounced and significant improvement of motor ability in both groups. Throughout the treatment period, continuous improvement of hand movements in the shaping tasks was observed in each patient. For example, increasingly smaller objects could be picked up faster and with progressively less effort. Furthermore, the patients' functional movement capabilities improved so that new tasks of daily living could be performed outside the laboratory and in the home environment after treatment. These 'new' real-world behaviours consisted of such activity of daily living (ADL) tasks as eating soup with a spoon, cutting meat, or combing the hair with the affected hand. Results further indicated that the affected hand was used more often and with better quality, which confirms that the improvements induced by training in the laboratory transferred into the home environment. Furthermore, the significant treatment effects were only observed for the trained hand (hand x treatment interaction) and remained unchanged for the follow-up period (main effect of treatment). Because the improvement was specific to the trained hand, the likelihood of non-specific placebo-type contributions to treatment outcome is low. Statistical analyses of the *subgroup* data further revealed that patients in the 3-hour group did improve with treatment. However, the therapy effects were considerably stronger in the 6-hour group. Since no differences existed in the baseline and pre-treatment values between the groups, the greater efficacy of the 6-hour training protocol was ascribed to the more intense training schedule. It might seem intuitively reasonable, and thus slightly unexciting, that a more intensive training protocol induced larger therapeutic effects. However, from a theoretical perspective, it is not obvious that a reduced CI-therapy protocol would retain any effectiveness. This is because both groups wore the constraint for the same amount of time and it is unclear how the forced-use induced by the constraint and the shaping training interact with regards to treatment efficacy. Furthermore, massed practice is an essential principle in CI-therapy, and until now little information about the relationship between the amount of training and the effectiveness of the treatment was available. Thus, the finding that 3 hours of treatment leads to clear, albeit limited improvement is interesting and important, since it shows that a significant and functionally relevant treatment effect can be obtained with a less demanding and less labour-intensive protocol.

A second step towards the clinical transfer of the CI therapy concept was taken by testing a training protocol that employed the shaping element only. The rationale for testing the efficacy of this approach was multifold. Firstly, there is good evidence that skill learning is mediated by discrete, experience-driven changes of the neural representations underlying the trained skill ([5, 16,17], Karni, 1998 #114, [61,63,65,76]). Secondly, such activity-dependent modulation of neural network outputs presumably plays a major role in the re-gain of function, and may be particularly relevant for the enhancement of recovery by rehabilitation treatments [8, 11,21,24,31,37,85,97]. Thirdly, the relevance of practice for the enhancement of long-term recovery is increasingly recognized [94]. Fourthly, the main objections to CI therapy appear to arise from concerns regarding the constraint element and the intensive one-to-one training. We therefore presumed that a shortened shaping-only training protocol would have a good chance to achieve reasonable treatment effects (since it presumably supports motor learning and brain plasticity) and, by accounting for the constraints given in standard care situations, is met with greater acceptance in the in-patient setting. The work was conducted in and with the existing resources of a rehabilitation clinic in Germany (Hegau Jugendwerk, Gailingen). Prior to the main experiment, pilot case studies were conducted in which various timescales, ranging from 1–2 hours of shaping training given daily for 1–4 weeks were tested [75]. The case studies clearly showed that the best 'trade off' between treatment benefits, resources and organisational limitations consisted of a 90 minute-per-day protocol given for a period of three weeks [73]). Subsequently, a clinical trial with chronic hemiparesis patients was conducted. The study employed an AB design that incorporated a 3-week baseline interval (A-phase) during which patients received physical therapy for 90 minutes each day in order to account for possible placebo-type effects arising from increased patient/therapist contact. Including the four-week follow-up period the study encompassed 12 consecutive weeks in total. Thirteen adults with chronic upper-limb hemiparesis (convenience sample) were enrolled in the program. Laboratory tests, 'real-world'-outcome measures, and standard scales were recorded on four occasions, before and after the A-phase, after the B-phase and at follow-up. Statistical data analysis of the various outcome parameters indicated that significant improvements occurred with the intervention (B-phase) but not during the A-phase. No changes were observed in the follow-up period, which indicates that the clinical benefits obtained in the B-phase were maintained after the intervention. Most importantly, we found that the treatment effects were observed only after the shaping training but not during the A-phase. Therefore, placebo-type 'more-treatment' effects can be excluded as a trivial explanation for the results. In addition, we were able to fully replicate the beneficial effects of the shaping training in the second study, which, for resource reasons, omitted the A-phase. The results obtained in these two studies are important in two ways. Firstly they show that the application of the shaping element improves motor performance in patients who have gone well beyond spontaneous recovery. Secondly, the protocol is practical in the clinical environment with respect to both, organizational concerns and resources.

It is important to note that the shaping-only regimen omitted the constraint element of CI therapy, due to the concerns outlined above. The data show that substantial clinical benefits can be achieved without constraint, but this does not imply the irrelevance of the constraint element for clinical outcome. Increased neural activity in the motor system is known to trigger functional reorganisation, and there is some evidence that CI therapy draws on neuroplasticity mechanisms to mediate enhanced motor control and the re-learning of movements with the hemiparetic limb. The combination of unaffected arm restriction and affected arm training further increases neural activity in the motor system, which most likely leads to stronger clinical effects. Furthermore, an experiment on the learned non-use phenomenon in high-functioning patients revealed that residual abilities are not fully incorporated in behavioural repertoire to a surprisingly high extent [74]. This suggests that the chain of behavioural ascendancy, which presumably underlies and maintains the conditioned behaviour of abated affected arm use, needs to be actively broken by means of behavioural intervention. This can be achieved by constraining the unaffected arm and the existing data suggest that this element of CIT supports the implementation of the recovered abilities into the behavioural repertoire. The latter is presumably crucial for the long-term benefit of the intervention and further studies will have to reveal, to what extend stable improvements can be obtained by shaping-only protocols.

## 7. Conclusion

Research activity in the fringe area of basic science and everyday clinical demands has contributed to a paradigm shift in neurological rehabilitation, not least by promoting theory-driven experimentation and the concept of evidence-based reasoning. New approaches such as CIT have been developed on the bases of new insights into the mechanisms of brain-plasticity, recovery and learning. These interventions are theoretically founded and subject to systematic experiments, which address both effect mechanisms and clinical outcome. The results consistently suggest that affected arm practice is the key to success in long-term recovery even in those patients who have long reached the plateau of recovery. Future research will have to aim to increase both applicability and acceptance in standard health care. One possible way forward will be to combine quantitative and qualitative research methods in order to identify practical hurdles, clinical reservations, and unqualified assumptions. These can subsequently be addressed experimentally, which will in turn allow an informed discussion and critical evaluation on the basis empirical data.

## Acknowledgement

Most of the data presented in this review has been collected at the Hegau Jugendwerk and I wish to express my thanks to Susanna Freivogel and all staff members for their support and enthusiasm in the various projects. I am further grateful to Professor Edward Taub, University of Alabama at Birmingham, USA, for his extensive support and scientific guidance.

The work was supported by a Career Establishment Grant awarded by the Medical Research Council, UK.
